# Predictive factors of acute sensorineural hearing loss in adult Japanese patients for clinical application by primary care doctors: a cross-sectional study

**DOI:** 10.1186/s12875-022-01830-8

**Published:** 2022-08-30

**Authors:** Toru Miwa, Tomoya Yamaguchi, Shin-ichiro Kita, Kazuto Osaka, Rie Kanai, Toshiki Maetani, Shin-ichi Kanemaru

**Affiliations:** 1 Department of Otolaryngology, Osaka Metropolitan University, 1-4-3 Asahimachi, Abeno-ku, Osaka, Japan; 2 Department of Otolaryngology-Head and Neck Surgery, Tazuke Kofukai Medical Research Institute Kitano Hospital, Osaka, Japan; 3 Department of Otolaryngology-Head and Neck Surgery, Kyoto University, Kyoto, Japan

**Keywords:** Acute sensorineural hearing loss, Diagnostic prediction, Multivariable logistic regression analysis

## Abstract

**Background:**

Several methods are used for hearing loss screening; however, their benefits are uncertain. In this study, we aimed to determine the predictive factors of acute sensorineural hearing loss for clinical application by primary care doctors.

**Methods:**

This retrospective, cross-sectional study included 365 patients with acute sensorineural hearing loss without prior therapy. The patients’ clinical data, demographic information, and medical histories were obtained, and they were asked about comorbidities. In addition, we assessed lifestyle factors such as stress level, alcohol consumption, marital status, and socioeconomic level. Logistic regression analysis was performed to investigate the diagnostic predictive ability of the selected factors associated with acute sensorineural hearing loss. The hearing levels of all patients were evaluated using pure tone audiometry.

**Results:**

We identified significant predictive factors for acute sensorineural hearing loss. The absence of hyperacusis was a predictive factor for sudden sensorineural hearing loss. Younger age, female sex, and marital status were predictive factors for acute low-tone hearing loss. High body mass index, high socioeconomic level, low alcohol consumption, high stress level, hyperacusis, and vertigo/dizziness were predictive factors for Ménière’s disease. High body mass index and ear fullness were predictive factors for perilymph fistula. Low stress level was a predictive factor for acoustic tumours.

**Conclusions:**

Our findings can be used to distinguish between the types of acute sensorineural hearing loss. Symptoms, physical status, and lifestyle factors identified during this study are useful markers for predicting acute sensorineural hearing loss occurrence.

**Supplementary Information:**

The online version contains supplementary material available at 10.1186/s12875-022-01830-8.

## Background

More than 30 million adults in the United States (approximately 15% of the population) experience hearing loss [1, 2]. Acute sensorineural hearing loss (ASHL) affects the conversion of mechanical sounds to neuroelectric signals in the inner ear or auditory nerve [1, 2]. Difficulty in hearing speech adversely affects social interactions and relationships. ASHL is associated with a decreased quality of life as well as the occurrence of dementia, depression, debility, delirium, falls, and death [1, 2]. Therefore, the development and implementation of modern therapeutic measures to eliminate ASHL are issues requiring urgent attention in otolaryngology.

ASHL includes the following types: sudden sensorineural hearing loss (SSNHL), acute low-tone hearing loss (ALHL), Ménière’s disease (MD), perilymph fistula, and retrocochlear disorders such as acoustic tumour [1–3]. Precise diagnoses are crucial for the successful treatment of ASHL, as treatment methods are disease- or condition-specific. However, an accurate diagnosis of ASHL is often challenging at the time of initial presentation because the initial symptoms of different types of ASHL are similar; therefore, it may be difficult to determine an appropriate treatment method and patient prognosis [1].

Previous studies have reported risk factors for ASHL [3–17]. However, in these studies, patients with different types of ASHL were compared with healthy individuals. Otolaryngologists diagnose each type of ASHL using pure tone audiometry; however, additional diagnostic tools are necessary for primary care doctors, unless they can make a referral for pure tone audiometry in the clinic and refer patients to otolaryngologists to accurately diagnose ASHL [1]. Although there are several approaches to screening for hearing loss, the benefits of each screening method remain uncertain. Identification of factors associated with ASHL may facilitate its early detection.

Therefore, this study aimed to determine the predictive factors of different types of ASHL to ensure accurate diagnosis and optimal treatment, with a focus on comparisons among ASHL patients. We did not assess the differences between ASHL patient and healthy individual groups.

## Methods

### Ethical approval

The Institutional Review Board of Kitano Hospital approved this study (approval number: 2001005; date of approval: 14 January, 2020) and waived the requirement for written informed consent because of the retrospective nature of the study. Moreover, the study was conducted in accordance with the principles of the 1964 Declaration of Helsinki and its later amendments or comparable ethical standards.

### Patients

This retrospective cross-sectional study included patients diagnosed with ASHL who visited our hospital between January 2014 and January 2019. The inclusion criteria were age ranging from 10 to 100 years and a diagnosis of unilateral ASHL. Patients who could not be adequately interviewed because of communication disorders and those with hearing loss attributable to other causes, such as brain damage, were excluded.

### Data collection

All patients underwent detailed clinical interviews conducted by physicians. Patient clinical data, demographic information, and medical history were obtained. The patients were asked about comorbidities, including hypertension (International Statistical Classification of Diseases and Related Health Problems version 10 [ICD10]: I10-15) and mental illness (ICD10: F00-99). Lifestyle factors, such as stress, alcohol consumption, marital status, and socioeconomic level, were assessed. All the participants underwent routine general physical examination, general otorhinolaryngological examination, and routine audiological and laboratory tests.

### Evaluation of hearing level

The hearing levels of all patients were evaluated using pure tone audiometry. Air conduction was assessed at frequencies of 125 Hz, 250 Hz, 500 Hz, 1 kHz, 2 kHz, 4 kHz, and 8 kHz. In patients with out-of-scale types of hearing loss, 5 dB was considered as a hearing level of 105 dB. For SSNHL, the average hearing level (average of five frequencies: 250–4000 Hz) was used according to the Japan Audiological Society guidelines [18]. For ALHL, the sum of three low-tone frequencies (125, 250, and 500 Hz) was used according to the Japan Audiological Society guidelines [18]. For MD, perilymph fistula, and acoustic tumour, both the average hearing level (average of five frequencies: 250–4000 Hz) and the sum of three low-tone frequencies (125, 250, and 500 Hz) were used, as the type of hearing loss varied according to the type of disease [19].

### Haematological evaluation

Haematological tests were performed in all patients to assess haemoglobin A1c (HbA1c) levels.

### Statistical analyses

Multivariable logistic regression analysis was performed using the incidence of each disease as the dependent variable to investigate the diagnostic predictive ability of selected factors associated with ASHL. The explanatory variables included objective symptoms, age, sex, body mass index (BMI), hypertension, HbA1c, mental illness, stress, Brinkman index score, alcohol consumption, insurance level (socioeconomic level), marital status, family history of hearing loss, subjective symptoms of tinnitus, ear fullness, hyperacusis, and vertigo/dizziness.

A model was created after confirming the variance inflation factor. Missing values were imputed using the random Forest method. There were no outliers during the analysis of primary or secondary outcomes. Interactions between parameters were evaluated for each disease. Statistical significance was set at *P*<0.05 (two-tailed). The evaluation results were considered not applicable if the calculated sample size after data collection was insufficient for statistical analysis.

All statistical analyses were performed using EZR (Saitama Medical Centre, Jichi Medical University, Saitama, Japan), which is a graphical user interface for R (The R Foundation for Statistical Computing, Vienna, Austria). Specifically, it is a modified version of the R commander designed to add statistical functions frequently used for biostatistics.

## Results

### Patient characteristics

**Fig. 1:**
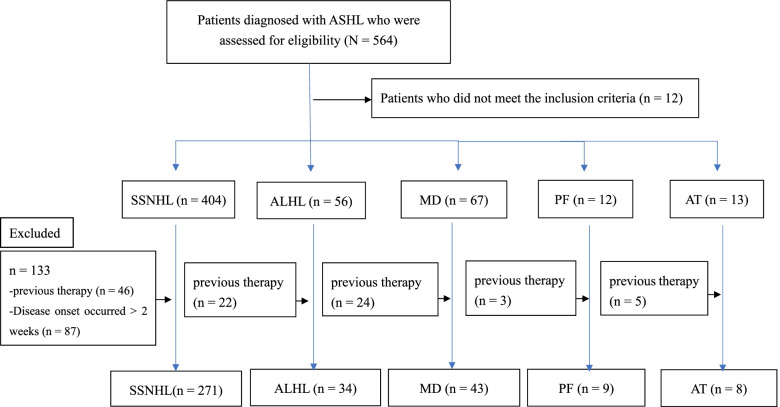
Patient selection process. SSNHL, sudden sensorineural hearing loss; ALHL, acute low-tone sensorineural hearing loss; MD, Ménière’s disease; PF, perilymph fistula; AT, acoustic tumour; ASHL, acute sensorineural hearing loss

In total, 564 patients were assessed. Among them, 12 patients who did not meet the diagnostic criteria for SSNHL, ALHL, MD, perilymph fistula, or acoustic tumour were excluded. We classified the patients into SSNHL, ALHL, MD, perilymph fistula, and acoustic tumour groups based on the type of ASHL diagnosed via hearing levels [18]. Among the 404 patients with SSNHL, 87 patients with a complaint of subjective hearing loss since more than 2 weeks before presentation were excluded. Additionally, we excluded 46, 22, 24, 3, and 5 patients with SSNHL, ALHL, MD, perilymph fistula, and acoustic tumour, respectively, who had previously visited other clinics or hospitals and received treatment (Fig. 1).

Finally, we included 365 patients in this study (176 men and 189 women). The average patient age was 55.2 years (standard deviation, ± 16.9 years). Other patient demographic characteristics are shown in Supplementary Table 1 [see Additional file 1].

### Predictive factors

Multivariable logistic regression analysis was performed, with the onset of each disease as the outcome variable. We found that hyperacusis was a predictive factor of SSNHL (odds ratio [OR]: 0.21; 95% confidence interval [CI]: 0.07–0.59; *P*=0.002) (Table 1), whereas age (OR: 0.95; 95% CI: 0.92–0.98; *P*=0.004), marital status (OR: 2.78; 95% CI: 1.12–6.90; *P*=0.02), and female sex (OR: 4.14; 95% CI: 1.47–11.6; *P*=0.007) were predictive factors for ALHL (Table 2). Alcohol consumption (OR: 0.63; 95% CI: 0.43–0.93; *P*=0.02), BMI (OR: 1.04; 95% CI: 1.00–1.09; *P*=0.04), hyperacusis (OR: 4.06; 95% CI: 1.39–11.9; *P*=0.01), socioeconomic level (OR: 0.53; 95% CI: 0.28–0.97; *P*=0.04), stress level (OR: 1.40; 95% CI: 1.03–1.88; *P*=0.02), and vertigo/dizziness (OR: 2.15; 95% CI: 1.11–4.16, *P*=0.02) were factors associated with MD (Table 3).

**Table 1:** Factors associated with sudden sensorineural hearing loss (*N*=365)

	Odds ratio [95% confidence interval]	***P***-value
**Age**	1.02 [0.99–1.04]	0.08
**Sex**	0.67 [0.37–1.21]	0.18
**Body mass index**	0.97 [0.93–1.01]	0.13
**Hypertension**	1.43 [0.71–2.88]	0.31
**Haemoglobin A1c**	1.40 [0.94–2.09]	0.09
**Mental illness**	1.21 [0.38–3.86]	0.74
**Stress**	0.94 [0.76–1.16]	0.56
**Brinkman index score**	1.00 [0.99–1.00]	0.83
**Alcohol consumption**	1.28 [0.98–1.67]	0.06
**Socioeconomic level**	1.07 [0.76–1.49]	0.68
**Marital status**	0.67 [0.38–1.18]	0.17
**Family history of hearing loss**	0.79 [0.36–1.71]	0.54
**Tinnitus**	1.53 [0.94–2.48]	0.08
**Ear fullness**	0.79 [0.51–1.21]	0.27
**Hyperacusis**	0.21 [0.07–0.59]	0.002**
**Vertigo/dizziness**	0.76 [0.45–1.27]	0.29

Null deviance: 416.44 with 364 degrees of freedom

Residual deviance: 370.18 with 346 degrees of freedom

Akaike’s information criterion value: 408.18

Interactions between the parameters are shown in Supplementary Table 4

**P<0.01

**Table 2:** Factors associated with acute low-tone sensorineural hearing loss (*N*=365)

	Odds ratio [95% confidence interval]	***P***-value
**Age**	0.95 [0.92–0.98]	0.004**
**Sex**	4.14 [1.47–11.60]	0.007**
**Body mass index**	0.97 [0.85–1.11]	0.72
**Hypertension**	0.53 [0.10–2.60]	0.43
**Haemoglobin A1c**	0.15 [0.02–1.01]	0.05
**Mental illness**	1.75 [0.36–8.25]	0.48
**Stress**	0.97 [0.69–1.37]	0.88
**Brinkman index score**	0.99 [0.99–1.00]	0.55
**Alcohol consumption**	0.83 [0.52–1.34]	0.45
**Socioeconomic level**	1.15 [0.62–2.11]	0.66
**Marital status**	2.78 [1.12–6.90]	0.02*
**Family history of hearing loss**	0.52 [0.11–2.53]	0.42
**Tinnitus**	0.60 [0.26–1.38]	0.23
**Ear fullness**	1.16 [0.59–2.29]	0.65
**Hyperacusis**	3.05 [0.73–12.60]	0.12
**Vertigo/dizziness**	0.52 [0.20–1.36]	0.18

Null deviance: 226.13 with 364 degrees of freedom

Residual deviance: 165.04 with 346 degrees of freedom

Akaike’s information criterion value: 203.04

Interactions between the parameters are shown in Supplementary Table 5

*P<0.05 **P<0.01

**Table 3:** Factors associated with Ménière’s disease (*N*=365)

	Odds ratio [95% confidence interval]	***P***-value
**Age**	1.02 [0.99–1.04]	0.22
**Sex**	0.94 [0.43–2.09]	0.89
**Body mass index**	1.04 [1.00–1.09]	0.04*
**Hypertension**	0.65 [0.25–1.67]	0.37
**Haemoglobin A1c**	0.88 [0.59–1.32]	0.54
**Mental illness**	0.37 [0.05–2.40]	0.29
**Stress**	1.40 [1.03–1.88]	0.02*
**Brinkman index score**	1.00 [1.00–1.01]	0.35
**Alcohol consumption**	0.63 [0.43–0.93]	0.02*
**Socioeconomic level**	0.53 [0.28–0.97]	0.04*
**Marital status**	1.15 [0.54–2.43]	0.72
**Family history of hearing loss**	1.66 [0.65–4.21]	0.29
**Tinnitus**	0.78 [0.41–1.48]	0.45
**Ear fullness**	0.92 [0.50–1.70]	0.80
**Hyperacusis**	4.06 [1.39–11.90]	0.01*
**Vertigo/dizziness**	2.15 [1.11–4.16]	0.02*

Null deviance: 264.65 with 364 degrees of freedom

Residual deviance: 233.30 with 346 degrees of freedom

Akaike’s information criterion value: 271.30

Interactions between the parameters are shown in Supplementary Table 6

*P<0.05

Variables associated with perilymph fistula occurrence included BMI (OR: 1.36; 95% CI: 1.08–1.71; *P*=0.01) and ear fullness (OR: 3.46; 95% CI: 1.02–11.7, *P*=0.04) (see Supplementary Table 2 in Additional File 1). Lastly, stress was a factor associated with acoustic tumour (OR: 0.44; 5% CI: 0.20–0.96; *P*=0.04) (see Supplementary Table 3 in Additional File 1).

## Discussion

This study explored the predictive factors of acute sensorineural hearing loss for clinical application by primary care doctors and found that patients with SSNHL were not likely to have hyperacusis compared to patients with other types of ASHL. Previous studies have reported that tinnitus, vertigo/dizziness, ear fullness, older age, smoking, cardiovascular diseases, hypertension, and diabetes mellitus are factors associated with SSNHL [2–4, 10–12]. Our results did not reveal many significant predictive factors for SSNHL, probably because we performed comparisons among patients with various types of ASHL rather than comparisons of patients with SSNHL with healthy participants. The predictive factors for SSNHL reported in previous studies [12, 20–28] is comparable in patients with other types of ASHL. Therefore, we found it difficult to distinguish between the predictive factors for other types of ASHL and SSNHL.

We found that patients with ALHL were more likely to be younger married women compared to patients with other types of ASHL. Similarly, previous studies [13–16] have revealed that younger age and female sex were factors associated with ALHL, which could be attributed to hormonal or genetic factors [13–16]. The prevalence of most anxiety disorders is approximately twice as high in women compared to men [29]. Our results showed an OR of mental illness in ALHL of 1.75, indicating a 1.44-fold and 4.72-fold increase compared to that in SSNHL and MD, respectively. In addition, the number of women was higher than that of men in our sample population of ALHL. These results indicate that women are more likely affected by anxiety, which strongly predisposes patients to ALHL [30]. Furthermore, unlike in Western countries, the marital status of patients in Japan might comprise a stress factor, especially for Japanese women, because of cultural norms such as traditional sex relationships [31, 32].

In our study, patients with MD were more likely to consume small amounts of alcohol and have higher BMI, stress levels, and self-care levels as well as a higher occurrence rates of vertigo/dizziness and hyperacusis than patients with other types of ASHL. The following factors associated with MD have been previously reported: older age, white race, female sex, high BMI, allergies, immune dysfunction, autonomic dysfunction, poor mental health, tinnitus, vertigo/dizziness, and ear fullness [5–7, 17, 33]. In addition, the results of an epidemiological survey conducted by the research team of the Ministry of Health, Labour, and Welfare in Japan showed that mental overload, physical overwork, and insufficient sleep associated with professional technical jobs performed by individuals with personality traits such as strictness and nervousness were likely to cause MD. High stress levels, especially due to job stressors, might be affected by personality traits and work conditions [34], resulting in autonomic dysfunction and immune system disruption [35–39]. Tyrrell et al. suggested that an intricate interaction between the autonomic and immune systems may contribute to the development of MD [17]. Our results mostly corroborated the results of the abovementioned epidemiological survey and those reported by Tyrrell et al. [17]. Furthermore, high stress levels increase plasma concentrations of the stress hormone vasopressin, its V2 receptor (V2R), and V2R-regulated water channel aquaporin-2 in the endolymphatic sac in patients with MD [40].

Our results suggest that patients with perilymph fistula are more likely to experience ear fullness than patients with other types of ASHL. A previous study reported vertigo/dizziness, ear fullness, and hyperacusis as factors associated with perilymph fistula [8]; however, we did not identify vertigo/dizziness and hyperacusis as predictive factors. This discrepancy may have occurred because we compared the predictive factors for various types of ASHL, whereas previous studies compared clinical and laboratory characteristics between patients and healthy participants. In addition, we showed that patients with acoustic tumours are more likely to experience low stress levels compared to patients with other types of ASHL. Previous studies have also reported that ear fullness and stress are factors associated with acoustic tumours [7, 9]. The present study indicated that a low stress level was the predictive factor for acoustic tumours. However, previous studies observed a strong positive correlation between cortisol and adrenocorticotropic hormone levels [7, 9]. This discrepancy may also have occurred because we compared the predictive factors for various types of ASHL, whereas previous studies compared clinical and laboratory characteristics between patients with ASHL and healthy participants.

### Study limitations

This study has several limitations. We did not compare clinical and laboratory profiles between patients with ASHL and healthy individuals. Therefore, further studies are warranted to perform these comparisons. Moreover, this was a retrospective cross-sectional study; hence, the causality of relationships could not be inferred. Furthermore, the stress levels of the participants had not been comprehensively assessed; stress level constitutes a potential factor associated with hearing loss; thus, the use of questionnaires should be considered in future studies. Although we identified potential factors associated with and comorbidities for ASHL for women and men, case-control and longitudinal cohort studies, with sufficient participant data collection, are warranted to investigate the mechanisms underlying the potential factors associated with ASHL. Lastly, we did not include conductive hearing loss in this study (i.e. cerumen impaction of the external ear canals and eardrums). Primary doctors may not examine the external canals. Further study is needed to clarify.

## Conclusions

Our diagnostic prediction analysis identified various predictors for each type of ASHL. Some comorbidities that were previously reported to be factors associated with ASHL were not found to be predictive factors in this study. Our findings can help primary care doctors distinguish between different types of ASHL without using an audiometer.

## Supplementary Information

**Additional file 1: Supplementary Table 1.** Demographics of patients with acute sensorineural hearing loss (*N*=365). **Supplementary Table 2.** Factors associated with perilymph fistula (*N*=365). **Supplementary Table 3.** Factors associated with acoustic tumour (*N*=365). **Supplementary Table 4.** *P* value for interaction between parameters for sudden sensorineural hearing loss. **Supplementary Table 5.** *P* value for interaction between parameters for acute low-tone sensorineural hearing loss. **Supplementary Table 6.** *P* value for interaction between parameters for Ménière’s disease. **Supplementary Table 7.** *P* value for interaction between parameters for perilymph fistula. **Supplementary Table 8.** *P* value for interaction between parameters for acoustic tumour.

## Data Availability

All data generated or analysed during this study are included in this published article and its supplementary material. All authors confirm that they had full access to the study data and take responsibility for the integrity of the data and the accuracy of the data analysis.

## Abbreviations

ALHL: Acute low-tone hearing loss
ASHL: Acute sensorineural hearing loss
BMI: Body mass index
CI: Confidence interval
HbA1c: Haemoglobin A1c
ICD: International Statistical Classification of Diseases and Related Health Problems
MD: Ménière’s disease
OR: Odds ratio
SSNHL: Sudden sensorineural hearing loss
