# Supplementary material for: Predictive factors of acute sensorineural hearing loss in adult Japanese patients for clinical application by primary care doctors: a cross-sectional study
Source: BMC Prim Care. 2022 Aug 30;23:219. doi: 10.1186/s12875-022-01830-8 (PMC9429655; doi:10.1186/s12875-022-01830-8)
Supplement: Supplementary file 1 — Additional file 1: Supplementary Table 1. Demographics of patients with acute sensorineural hearing loss (N=365). Supplementary Table 2. Factors associated with perilymph fistula (N=365). Supplementary Table 3. Factors associated with acoustic tumour (N=365). Supplementary Table 4. P value for interaction between parameters for sudden sensorineural hearing loss. Supplementary Table 5. P value for interaction between parameters for acute low-tone sensorineural hearing loss. Supplementary Table 6. P value for interaction between parameters for Ménière’s disease. Supplementary Table 7. P value for interaction between parameters for perilymph fistula. Supplementary Table 8. P value for interaction between parameters for acoustic tumour. [file 12875_2022_1830_MOESM1_ESM.docx]

# Additional file 1

# Supplementary Table 1. Demographics of patients with acute sensorineural hearing loss (N=365)

| **Variables** | | **SSNHL (n=271)** | | | | **ALHL (n=34)** | | | | **MD (n=43)** | | | | **PF (n=9)** | | | | **AT (n=8)** | | | |
| --- | --- | --- | --- | --- | --- | --- | --- | --- | --- | --- | --- | --- | --- | --- | --- | --- | --- | --- | --- | --- | --- |
| **Age range, years (mean±SD)** | **Sum** | 10–88 (56.6±16.6) | | | | 22–68 (43.2±12.6) | | | | 18–92 (54.6±17.2) | | | | 40–69 (54.6±11.8) | | | | 26–84 (55.3±16.8) | | | |
|  | **Male** | 55.4 | | | | 38.8 | | | | 53.6 | | | | 56.0 | | | | 60.2 | | | |
|  | **Female** | 58.0 | | | | 44.8 | | | | 55.3 | | | | 51.6 | | | | 50.5 | | | |
| **Sex, n** | **Male** | 139 | | | | 8 | | | | 19 | | | | 6 | | | | 4 | | | |
|  | **Female** | 132 | | | | 26 | | | | 24 | | | | 3 | | | | 4 | | | |
| **BMI** | **Sum** | 23.6±7.1 | | | | 22.5±2.8 | | | | 24.9±4.2 | | | | 25.9±2.9 | | | | 22.0±2.6 | | | |
|  | **Male** | 23.9 | | | | 24.9 | | | | 25.1 | | | | 24.9 | | | | 22.7 | | | |
|  | **Female** | 23.2 | | | | 21.8 | | | | 24.7 | | | | 27.6 | | | | 21.4 | | | |
| **HT, n (%)** | **Sum** | 83 (30.2) | | | | 2 (5.8) | | | | 8 (18.6) | | | | 3 (33.3) | | | | 2 (25) | | | |
|  | **Male** | 44 | | | | 1 | | | | 2 | | | | 2 | | | | 0 | | | |
|  | **Female** | 39 | | | | 1 | | | | 6 | | | | 1 | | | | 2 | | | |
| **HbA1c (%)** | **Sum** | 5.9±1.0 | | | | 5.4±0.2 | | | | 5.8±1.0 | | | | 5.7±0.6 | | | | 5.4±0.6 | | | |
|  | **Male** | 5.9 | | | | 5.4 | | | | 5.8 | | | | 5.7 | | | | 5.4 | | | |
|  | **Female** | 6.0 | | | | 5.4 | | | | 5.8 | | | | 5.7 | | | | 5.5 | | | |
| **Mental disorders, n (%)** | **Sum** | 16(5.9) | | | | 3 (8.8) | | | | 2 (4.6) | | | | 0 (0) | | | | 1 (12.5) | | | |
|  | **Male** | 9 | | | | 1 | | | | 0 | | | | 0 | | | | 0 | | | |
|  | **Female** | 7 | | | | 2 | | | | 2 | | | | 0 | | | | 1 | | | |
| **Stress level (low, 0; high, 3)** | **Sum** | 87 | 29 | 50 | 105 | 5 | 7 | 11 | 11 | 5 | 8 | 12 | 18 | 4 | 1 | 1 | 3 | 6 | 0 | 1 | 1 |
|  | **Male** | 48 | 15 | 22 | 54 | 0 | 2 | 5 | 1 | 1 | 5 | 3 | 10 | 3 | 1 | 1 | 1 | 3 | 0 | 1 | 0 |
|  | **Female** | 39 | 14 | 28 | 51 | 5 | 5 | 6 | 10 | 4 | 3 | 9 | 8 | 1 | 0 | 0 | 2 | 3 | 0 | 0 | 1 |
| **BI** | **Sum** | 225.1±419.6 | | | | 77.3±149.5 | | | | 234.3±537.4 | | | | 260.0±420.0 | | | | 66.8±122.3 | | | |
|  | **Male** | 360.2 | | | | 135.5 | | | | 448.2 | | | | 390 | | | | 90 | | | |
|  | **Female** | 82.8 | | | | 59.5 | | | | 65 | | | | 0 | | | | 43.7 | | | |
| **Alcohol consumption, n (low, 0; high, 3)** | **Sum** | 110 | 68 | 34 | 59 | 16 | 10 | 7 | 1 | 16 | 21 | 3 | 3 | 4 | 2 | 0 | 3 | 1 | 3 | 2 | 2 |
|  | **Male** | 41 | 33 | 21 | 44 | 1 | 4 | 2 | 1 | 4 | 9 | 3 | 3 | 1 | 2 | 0 | 3 |  |  |  |  |
|  | **Female** | 69 | 35 | 13 | 15 | 15 | 6 | 5 | 0 | 12 | 12 | 0 | 0 | 3 | 0 | 0 | 0 |  |  |  |  |
| **Socioeconomic level, n (high, 0; low, 3)** | **Sum** | 195 | 26 | 25 | 25 | 27 | 6 | 0 | 1 | 35 | 5 | 2 | 1 | 4 | 2 | 0 | 3 | 7 | 0 | 1 | 0 |
|  | **Male** | 105 | 11 | 8 | 15 | 7 | 1 | 0 | 0 | 16 | 2 | 1 | 0 | 3 | 1 | 0 | 2 |  |  |  |  |
|  | **Female** | 90 | 15 | 17 | 10 | 20 | 5 | 0 | 1 | 19 | 3 | 1 | 1 | 1 | 1 | 0 | 1 |  |  |  |  |
| **Marital status, n (%)** | **Sum** | 168 (61.9) | | | | 22 (64.7) | | | | 28 (65.1) | | | | 6 (66.6) | | | | 3 (37.5) | | | |
|  | **Male** | 94 | | | | 5 | | | | 12 | | | | 4 | | | | 2 | | | |
|  | **Female** | 74 | | | | 17 | | | | 16 | | | | 2 | | | | 1 | | | |
| **Family history, n (%)** | **Sum** | 33 (12.1) | | | | 2 (5.8) | | | | 8 (18.6) | | | | 2 (22.2) | | | | 1 (12.5) | | | |
|  | **Male** | 14 | | | | 1 | | | | 4 | | | | 1 | | | | 0 | | | |
|  | **Female** | 19 | | | | 1 | | | | 4 | | | | 1 | | | | 1 | | | |
| **Tinnitus, n (%)** | **Sum** | 151 (5546.7) | | | | 14 (41.1) | | | | 20 (46.5) | | | | 4 (44.4) | | | | 3 (37.5) | | | |
|  | **Male** | 69 | | | | 5 | | | | 8 | | | | 3 | | | | 1 | | | |
|  | **Female** | 72 | | | | 9 | | | | 12 | | | | 1 | | | | 2 | | | |
| **Ear fullness, n (%)** | **Sum** | 119 (43.9) | | | | 19 (55.8) | | | | 18 (41.8) | | | | 5 (55.5) | | | | 3 (37.5) | | | |
|  | **Male** | 46 | | | | 3 | | | | 9 | | | | 4 | | | | 2 | | | |
|  | **Female** | 61 | | | | 16 | | | | 9 | | | | 1 | | | | 1 | | | |
| **Hyperacusis, n (%)** | **Sum** | 6 (2.2) | | | | 4 (11.7) | | | | 5 (11.6) | | | | 1 (11.1) | | | | 0 (0) | | | |
|  | **Male** | 0 | | | | 1 | | | | 4 | | | | 1 | | | | 0 | | | |
|  | **Female** | 6 | | | | 3 | | | | 1 | | | | 0 | | | | 0 | | | |
| **Vertigo/dizziness, n (%)** | **Sum** | 61 (22.5) | | | | 8 (23.5) | | | | 16 (37.2) | | | | 3 (33.3) | | | | 2 (25) | | | |
|  | **Male** | 26 | | | | 1 | | | | 4 | | | | 2 | | | | 1 | | | |
|  | **Female** | 35 | | | | 7 | | | | 11 | | | | 1 | | | | 1 | | | |
| **Average hearing level at 250–4000 Hz, dB, mean±SD** | **Sum** | 60.3±26.6 | | | | NA | | | | 37.4±18.1 | | | | 49.0±20.3 | | | | 69.0±28.7 | | | |
|  | **Male** | 59.2 | | | | NA | | | | 36.0 | | | | 47.6 | | | | 50.0 | | | |
|  | **Female** | 61.5 | | | | NA | | | | 38.6 | | | | 51.6 | | | | 88.0 | | | |
| **Sum of hearing level (125, 250, 500 Hz), dB, mean±SD** | **Sum** | NA | | | | 138.3±44.3 | | | | 132.2±50.3 | | | | NA | | | | 190.6±88.7 | | | |
|  | **Male** | NA | | | | 146.2 | | | | 127.8 | | | | NA | | | | 125.0 | | | |
|  | **Female** | NA | | | | 135.9 | | | | 135.6 | | | | NA | | | | 256.2 | | | |

Abbreviations: BMI, body mass index; HT, hypertension; HbA1c, haemoglobin A1c; BI, Brinkman index; SSNHL, sudden sensorineural hearing loss; ALHL, acute low-tone sensorineural hearing loss; MD, Ménière’s disease; PF, perilymph fistula; AT, acoustic tumour; NA, not applicable; SD, standard deviation.

Supplementary Table 2. Factors associated with perilymph fistula (N=365)

|  | Odds ratio [95% confidence interval] | *P-*value |
| --- | --- | --- |
| Age | 0.99 [0.93–1.05] | 0.80 |
| Sex | 0.28 [0.04–1.73] | 0.17 |
| Body mass index | 1.36 [1.08–1.71] | 0.01* |
| Hypertension | 0.88 [0.13–5.94] | 0.89 |
| Haemoglobin A1c | 0.57 [0.15–2.10] | 0.40 |
| Mental illness | 0.0004 [0.0001–0.0002] | 0.98 |
| Stress | 0.92 [0.47–1.80] | 0.82 |
| Brinkman index score | 1.00 [0.99–1.00] | 0.87 |
| Alcohol consumption | 0.79 [0.37–1.71] | 0.56 |
| Socioeconomic level | 1.89 [0.99–3.62] | 0.05 |
| Marital status | 1.03 [0.16–6.23] | 0.97 |
| Family history of hearing loss | 2.40 [0.29–19.30] | 0.41 |
| Tinnitus | 0.87 [0.24–3.16] | 0.84 |
| Ear fullness | 3.46 [1.02–11.70] | 0.04* |
| Hyperacusis | 5.07 [0.33–78.00] | 0.24 |
| Vertigo/dizziness | 2.00 [0.50–7.88] | 0.32 |

Null deviance: 84.42 with 364 degrees of freedom.

Residual deviance: 62.01 with 346 degrees of freedom.

Akaike’s information criterion value: 100.01.

**P*<0.05

Supplementary Table 3. Factors associated with acoustic tumour (N=365)

|  | Odds ratio [95% confidence interval] | *P-*value |
| --- | --- | --- |
| Age | 1.05 [0.98–1.13] | 0.15 |
| Sex | 0.45 [0.07–2.83] | 0.39 |
| Body mass index | 0.91 [0.67–1.25] | 0.58 |
| Hypertension | 0.54 [0.06–4.46] | 0.57 |
| Haemoglobin A1c | 0.19 [0.009–4.01] | 0.28 |
| Mental illness | 6.76 [0.40–112.01] | 0.18 |
| Stress | 0.44 [0.20–0.96] | 0.04* |
| Brinkman index score | 0.99 [0.99–1.00] | 0.25 |
| Alcohol consumption | 2.08 [0.91–4.72] | 0.07 |
| Socioeconomic level | 0.62 [0.17–2.21] | 0.46 |
| Marital status | 0.19 [0.02–1.45] | 0.11 |
| Family history of hearing loss | 1.73 [0.14–20.60] | 0.66 |
| Tinnitus | 0.51 [0.12–2.10] | 0.35 |
| Ear fullness | 0.81 [0.18–3.50] | 0.77 |
| Hyperacusis | 0.00001 [0.0001–0.0004] | 0.99 |
| Vertigo/dizziness | 0.75 [0.11–4.84] | 0.76 |

Null deviance: 76.95 with 364 degrees of freedom.

Residual deviance: 52.39 with 346 degrees of freedom.

Akaike’s information criterion value: 90.39.

**P*<0.05

Supplementary Table 4. P value for interaction between parameters for sudden sensorineural hearing loss

| P value for interaction | **Age** | **Sex** | **Body mass index** | **Hypertension** | **Haemoglobin A1c** | **Mental illness** | **Stress** | **Brinkman index score** | **Alcohol consumption** | **Socioeconomic level** | **Marital status** | **Family history of hearing loss** | **Tinnitus** | **Ear fullness** | **Hyperacusis** | **Vertigo/dizziness** |
| --- | --- | --- | --- | --- | --- | --- | --- | --- | --- | --- | --- | --- | --- | --- | --- | --- |
| **Age** |  |  |  |  |  |  |  |  |  |  |  |  |  |  |  |  |
| **Sex** | 0.46 |  |  |  |  |  |  |  |  |  |  |  |  |  |  |  |
| **Body mass index** | 0.12 | 0.98 |  |  |  |  |  |  |  |  |  |  |  |  |  |  |
| **Hypertension** | 0.77 | 0.53 | 0.34 |  |  |  |  |  |  |  |  |  |  |  |  |  |
| **Haemoglobin A1c** | 0.59 | 0.38 | 0.56 | 0.24 |  |  |  |  |  |  |  |  |  |  |  |  |
| **Mental illness** | 0.7 | 0.23 | 0.33 | 0.34 | 0.54 |  |  |  |  |  |  |  |  |  |  |  |
| **Stress** | 0.86 | 0.86 | 0.56 | 0.77 | 0.56 | 0.56 |  |  |  |  |  |  |  |  |  |  |
| **Brinkman index score** | 0.56 | 0.68 | 0.43 | 0.65 | 0.23 | 0.54 | 0.56 |  |  |  |  |  |  |  |  |  |
| **Alcohol consumption** | 0.67 | 0.29 | 0.3 | 0.78 | 0.45 | 0.33 | 0.33 | 0.56 |  |  |  |  |  |  |  |  |
| **Socioeconomic level** | 0.89 | 0.56 | 0.12 | 0.34 | 0.56 | 0.21 | 0.67 | 0.55 | 0.67 |  |  |  |  |  |  |  |
| **Marital status** | 0.27 | 0.28 | 0.23 | 0.45 | 0.6 | 0.18 | 0.77 | 0.32 | 0.58 | 0.87 |  |  |  |  |  |  |
| **Family history of hearing loss** | 0.74 | 0.3 | 0.56 | 0.45 | 0.75 | 0.57 | 0.22 | 0.3 | 0.55 | 0.76 | 0.33 |  |  |  |  |  |
| **Tinnitus** | 0.51 | 0.78 | 0.23 | 0.55 | 0.33 | 0.56 | 0.26 | 0.22 | 0.28 | 0.28 | 0.22 | 0.46 |  |  |  |  |
| **Ear fullness** | 0.95 | 0.23 | 0.57 | 0.21 | 0.24 | 0.88 | 0.19 | 0.19 | 0.47 | 0.35 | 0.1 | 0.67 | 0.69 |  |  |  |
| **Hyperacusis** | 0.99 | 0.15 | 0.88 | 0.22 | 0.23 | 0.67 | 0.3 | 0.87 | 0.78 | 0.78 | 0.89 | 0.66 | 0.98 | 0.81 |  |  |
| **Vertigo/dizziness** | 0.73 | 0.6 | 0.55 | 0.56 | 0.29 | 0.93 | 0.56 | 0.78 | 0.89 | 0.3 | 0.44 | 0.22 | 0.62 | 0.22 | 0.87 |  |

Supplementary Table 5. P value for interaction between parameters for acute low-tone sensorineural hearing loss

| P value for interaction | **Age** | **Sex** | **Body mass index** | **Hypertension** | **Haemoglobin A1c** | **Mental illness** | **Stress** | **Brinkman index score** | **Alcohol consumption** | **Socioeconomic level** | **Marital status** | **Family history of hearing loss** | **Tinnitus** | **Ear fullness** | **Hyperacusis** | **Vertigo/dizziness** |
| --- | --- | --- | --- | --- | --- | --- | --- | --- | --- | --- | --- | --- | --- | --- | --- | --- |
| **Age** |  |  |  |  |  |  |  |  |  |  |  |  |  |  |  |  |
| **Sex** | 0.41 |  |  |  |  |  |  |  |  |  |  |  |  |  |  |  |
| **Body mass index** | 0.13 | 0.57 |  |  |  |  |  |  |  |  |  |  |  |  |  |  |
| **Hypertension** | 0.88 | 0.23 | 0.57 |  |  |  |  |  |  |  |  |  |  |  |  |  |
| **Haemoglobin A1c** | 0.59 | 0.47 | 0.9 | 0.33 |  |  |  |  |  |  |  |  |  |  |  |  |
| **Mental illness** | 0.78 | 0.22 | 0.45 | 0.45 | 0.87 |  |  |  |  |  |  |  |  |  |  |  |
| **Stress** | 0.99 | 0.86 | 0.55 | 0.58 | 0.33 | 0.56 |  |  |  |  |  |  |  |  |  |  |
| **Brinkman index score** | 0.55 | 0.78 | 0.88 | 0.77 | 0.2 | 0.54 | 0.5 |  |  |  |  |  |  |  |  |  |
| **Alcohol consumption** | 0.76 | 0.33 | 0.12 | 0.21 | 0.56 | 0.33 | 0.56 | 0.74 |  |  |  |  |  |  |  |  |
| **Socioeconomic level** | 0.99 | 0.57 | 0.22 | 0.23 | 0.55 | 0.21 | 0.55 | 0.56 | 0.56 |  |  |  |  |  |  |  |
| **Marital status** | 0.37 | 0.33 | 0.38 | 0.56 | 0.78 | 0.18 | 0.68 | 0.22 | 0.47 | 0.27 |  |  |  |  |  |  |
| **Family history of hearing loss** | 0.28 | 0.78 | 0.67 | 0.45 | 0.6 | 0.57 | 0.78 | 0.21 | 0.21 | 0.46 | 0.28 |  |  |  |  |  |
| **Tinnitus** | 0.65 | 0.8 | 0.22 | 0.87 | 0.66 | 0.56 | 0.99 | 0.19 | 0.89 | 0.22 | 0.88 | 0.47 |  |  |  |  |
| **Ear fullness** | 0.88 | 0.22 | 0.57 | 0.98 | 0.23 | 0.88 | 0.72 | 0.79 | 0.46 | 0.2 | 0.37 | 0.67 | 0.81 |  |  |  |
| **Hyperacusis** | 0.1 | 0.14 | 0.8 | 0.87 | 0.44 | 0.67 | 0.34 | 0.99 | 0.35 | 0.69 | 0.47 | 0.77 | 0.72 | 0.63 |  |  |
| **Vertigo/dizziness** | 0.38 | 0.55 | 0.65 | 0.22 | 0.29 | 0.93 | 0.67 | 0.74 | 0.88 | 0.78 | 0.45 | 0.89 | 0.78 | 0.21 | 0.88 |  |

Supplementary Table 6. P value for interaction between parameters for Ménière’s disease

| P value for interaction | **Age** | **Sex** | **Body mass index** | **Hypertension** | **Haemoglobin A1c** | **Mental illness** | **Stress** | **Brinkman index score** | **Alcohol consumption** | **Socioeconomic level** | **Marital status** | **Family history of hearing loss** | **Tinnitus** | **Ear fullness** | **Hyperacusis** | **Vertigo/dizziness** |
| --- | --- | --- | --- | --- | --- | --- | --- | --- | --- | --- | --- | --- | --- | --- | --- | --- |
| **Age** |  |  |  |  |  |  |  |  |  |  |  |  |  |  |  |  |
| **Sex** | 0.86 |  |  |  |  |  |  |  |  |  |  |  |  |  |  |  |
| **Body mass index** | 0.24 | 0.57 |  |  |  |  |  |  |  |  |  |  |  |  |  |  |
| **Hypertension** | 0.12 | 0.23 | 0.47 |  |  |  |  |  |  |  |  |  |  |  |  |  |
| **Haemoglobin A1c** | 0.67 | 0.35 | 0.27 | 0.3 |  |  |  |  |  |  |  |  |  |  |  |  |
| **Mental illness** | 0.77 | 0.2 | 0.91 | 0.27 | 0.22 |  |  |  |  |  |  |  |  |  |  |  |
| **Stress** | 0.76 | 0.47 | 0.37 | 0.66 | 0.39 | 0.57 |  |  |  |  |  |  |  |  |  |  |
| **Brinkman index score** | 0.29 | 0.48 | 0.73 | 0.44 | 0.39 | 0.54 | 0.27 |  |  |  |  |  |  |  |  |  |
| **Alcohol consumption** | 0.72 | 0.88 | 0.52 | 0.68 | 0.67 | 0.3 | 0.57 | 0.27 |  |  |  |  |  |  |  |  |
| **Socioeconomic level** | 0.38 | 0.89 | 0.33 | 0.98 | 0.77 | 0.38 | 0.47 | 0.82 | 0.48 |  |  |  |  |  |  |  |
| **Marital status** | 0.22 | 0.98 | 0.46 | 0.32 | 0.89 | 0.19 | 0.68 | 0.37 | 0.59 | 0.39 |  |  |  |  |  |  |
| **Family history of hearing loss** | 0.1 | 0.36 | 0.48 | 0.71 | 0.7 | 0.29 | 0.33 | 0.21 | 0.21 | 0.38 | 0.29 |  |  |  |  |  |
| **Tinnitus** | 0.48 | 0.47 | 0.87 | 0.48 | 0.57 | 0.48 | 0.31 | 0.91 | 0.29 | 0.44 | 0.37 | 0.48 |  |  |  |  |
| **Ear fullness** | 0.47 | 0.58 | 0.73 | 0.89 | 0.37 | 0.77 | 0.21 | 0.73 | 0.11 | 0.28 | 0.37 | 0.67 | 0.37 |  |  |  |
| **Hyperacusis** | 0.92 | 0.56 | 0.88 | 0.37 | 0.33 | 0.67 | 0.38 | 0.33 | 0.29 | 0.93 | 0.38 | 0.87 | 0.37 | 0.37 |  |  |
| **Vertigo/dizziness** | 0.77 | 0.89 | 0.27 | 0.82 | 0.29 | 0.8 | 0.29 | 0.55 | 0.37 | 0.77 | 0.57 | 0.99 | 0.48 | 0.66 | 0.67 |  |

Supplementary Table 7. P value for interaction between parameters for perilymph fistula

| P value for interaction | **Age** | **Sex** | **Body mass index** | **Hypertension** | **Haemoglobin A1c** | **Mental illness** | **Stress** | **Brinkman index score** | **Alcohol consumption** | **Socioeconomic level** | **Marital status** | **Family history of hearing loss** | **Tinnitus** | **Ear fullness** | **Hyperacusis** | **Vertigo/dizziness** |
| --- | --- | --- | --- | --- | --- | --- | --- | --- | --- | --- | --- | --- | --- | --- | --- | --- |
| **Age** |  |  |  |  |  |  |  |  |  |  |  |  |  |  |  |  |
| **Sex** | 0.85 |  |  |  |  |  |  |  |  |  |  |  |  |  |  |  |
| **Body mass index** | 0.23 | 0.27 |  |  |  |  |  |  |  |  |  |  |  |  |  |  |
| **Hypertension** | 0.22 | 0.37 | 0.57 |  |  |  |  |  |  |  |  |  |  |  |  |  |
| **Haemoglobin A1c** | 0.67 | 0.47 | 0.21 | 0.47 |  |  |  |  |  |  |  |  |  |  |  |  |
| **Mental illness** | 0.77 | 0.30 | 0.19 | 0.37 | 0.28 |  |  |  |  |  |  |  |  |  |  |  |
| **Stress** | 0.66 | 0.49 | 0.91 | 0.38 | 0.39 | 0.53 |  |  |  |  |  |  |  |  |  |  |
| **Brinkman index score** | 0.30 | 0.42 | 0.23 | 0.10 | 0.40 | 0.18 | 0.21 |  |  |  |  |  |  |  |  |  |
| **Alcohol consumption** | 0.77 | 0.17 | 0.38 | 0.11 | 0.48 | 0.38 | 0.37 | 0.29 |  |  |  |  |  |  |  |  |
| **Socioeconomic level** | 0.38 | 0.21 | 0.29 | 0.29 | 0.87 | 0.49 | 0.55 | 0.29 | 0.39 |  |  |  |  |  |  |  |
| **Marital status** | 0.29 | 0.48 | 0.58 | 0.38 | 0.81 | 0.84 | 0.39 | 0.48 | 0.48 | 0.32 |  |  |  |  |  |  |
| **Family history of hearing loss** | 0.19 | 0.29 | 0.93 | 0.37 | 0.70 | 0.91 | 0.39 | 0.38 | 0.88 | 0.29 | 0.39 |  |  |  |  |  |
| **Tinnitus** | 0.48 | 0.92 | 0.28 | 0.28 | 0.37 | 0.88 | 0.19 | 0.28 | 0.28 | 0.38 | 0.28 | 0.39 |  |  |  |  |
| **Ear fullness** | 0.67 | 0.38 | 0.37 | 0.47 | 0.57 | 0.73 | 0.21 | 0.28 | 0.12 | 0.42 | 0.34 | 0.55 | 0.22 |  |  |  |
| **Hyperacusis** | 0.17 | 0.56 | 0.49 | 0.37 | 0.73 | 0.28 | 0.32 | 0.28 | 0.20 | 0.48 | 0.38 | 0.87 | 0.64 | 0.23 |  |  |
| **Vertigo/dizziness** | 0.38 | 0.29 | 0.99 | 0.82 | 0.71 | 0.37 | 0.21 | 0.59 | 0.33 | 0.44 | 0.27 | 0.91 | 0.58 | 0.49 | 0.21 |  |

Supplementary Table 8. P value for interaction between parameters for acoustic tumor

| P value for interaction | **Age** | **Sex** | **Body mass index** | **Hypertension** | **Haemoglobin A1c** | **Mental illness** | **Stress** | **Brinkman index score** | **Alcohol consumption** | **Socioeconomic level** | **Marital status** | **Family history of hearing loss** | **Tinnitus** | **Ear fullness** | **Hyperacusis** | **Vertigo/dizziness** |
| --- | --- | --- | --- | --- | --- | --- | --- | --- | --- | --- | --- | --- | --- | --- | --- | --- |
| **Age** |  |  |  |  |  |  |  |  |  |  |  |  |  |  |  |  |
| **Sex** | 0.24 |  |  |  |  |  |  |  |  |  |  |  |  |  |  |  |
| **Body mass index** | 0.33 | 0.37 |  |  |  |  |  |  |  |  |  |  |  |  |  |  |
| **Hypertension** | 0.21 | 0.89 | 0.37 |  |  |  |  |  |  |  |  |  |  |  |  |  |
| **Haemoglobin A1c** | 0.39 | 0.43 | 0.48 | 0.27 |  |  |  |  |  |  |  |  |  |  |  |  |
| **Mental illness** | 0.84 | 0.33 | 0.21 | 0.20 | 0.29 |  |  |  |  |  |  |  |  |  |  |  |
| **Stress** | 0.99 | 0.72 | 0.48 | 0.59 | 0.39 | 0.52 |  |  |  |  |  |  |  |  |  |  |
| **Brinkman index score** | 0.83 | 0.47 | 0.32 | 0.55 | 0.33 | 0.53 | 0.23 |  |  |  |  |  |  |  |  |  |
| **Alcohol consumption** | 0.32 | 0.84 | 0.33 | 0.47 | 0.38 | 0.29 | 0.38 | 0.73 |  |  |  |  |  |  |  |  |
| **Socioeconomic level** | 0.20 | 0.41 | 0.48 | 0.47 | 0.40 | 0.20 | 0.47 | 0.88 | 0.41 |  |  |  |  |  |  |  |
| **Marital status** | 0.49 | 0.27 | 0.68 | 0.47 | 0.48 | 0.10 | 0.36 | 0.49 | 0.37 | 0.29 |  |  |  |  |  |  |
| **Family history of hearing loss** | 0.20 | 0.38 | 0.98 | 0.72 | 0.47 | 0.33 | 0.33 | 0.40 | 0.49 | 0.38 | 0.22 |  |  |  |  |  |
| **Tinnitus** | 0.39 | 0.47 | 0.73 | 0.43 | 0.82 | 0.50 | 0.55 | 0.37 | 0.67 | 0.34 | 0.3 | 0.42 |  |  |  |  |
| **Ear fullness** | 0.48 | 0.47 | 0.47 | 0.80 | 0.84 | 0.82 | 0.37 | 0.72 | 0.68 | 0.28 | 0.83 | 0.63 | 0.37 |  |  |  |
| **Hyperacusis** | 0.38 | 0.38 | 0.57 | 0.38 | 0.36 | 0.49 | 0.3 | 0.39 | 0.30 | 0.63 | 0.32 | 0.22 | 0.27 | 0.81 |  |  |
| **Vertigo/dizziness** | 0.99 | 0.82 | 0.77 | 0.33 | 0.33 | 0.28 | 0.39 | 0.25 | 0.81 | 0.97 | 0.28 | 0.38 | 0.37 | 0.22 | 0.28 |  |
